# Un adénofibrome kystisé se révélant par un tableau de mastite infectieuse en post partum

**DOI:** 10.11604/pamj.2017.28.148.13491

**Published:** 2017-10-17

**Authors:** Meryem Belmajdoub, Sofia Jayi

**Affiliations:** 1 Service de Gynécologie Obstétrique II, CHU Hassan II, Fès, Maroc

**Keywords:** Mastite infectieuse, post partum, adénofibrome kystisé, Infectious mastitis, postpartum, cystic fibroadenoma

## Abstract

We report the case of Ms. A.A, a primiparous woman aged 21 years presenting (4 months after delivery) with an increase in left breast volume occurred 1 week after delivery associated with fever. The patient was put on amoxicillin, protected by suspending breast-feeding for several weeks without improvement. Clinical examination showed febrile patient with a temperature of 39°, an increase in left breast volume, inflammatory signs especially at the level of internal quadrants (A). Palpation showed a painful, hot collection adherent to the skin, measuring 16 cm, at the level of the internal quadrants, invading the external quadrants, without axillary adenopathies, suggesting breast abscess. Ultrasound (B) showed voluminous solidocystic predominantly liquid echogenic mass with thick walls and buds up to 46 mm, classified as ACR4. Puncture biopsy collected grayish green liquid, sent for bacteriological examination; antibiotic therapy with quinolones was started. Follow up of patient, after 3 days, showed reduction of the inflammatory signs and bacteriological examination of the liquid found no germ. After 15 days of antibiotic therapy, the inflammatory signs had disappeared and the volume of the mass had reduced enough, hence the indication for cystectomy. Cystectomy with simple enucleation was performed (C); surprisingly, the histological examination showed cystic fibroadenoma (D).

## Image en médecine

Nous rapportons le cas de Mme A.A, âgée de 21 ans primigeste, consulte chez nous (à 4 mois du post-partum de l'accouchement) pour une augmentation du volume du sein Gauche débutant une semaine après l'accouchement dans un contexte fébrile, suite auquel la patiente a été mise sous amoxicilline protégée pendant plusieurs semaines avec arrêt de l'allaitement mais sans amélioration. L'examen clinique a trouvé une patiente fébrile à 39°, un sein Gauche augmenté de volume avec des signes inflammatoires surtout au niveau des quadrants internes (A). La palpation a trouvé une collection de 16 cm, Douloureuse, chaude et adhérente à la peau, siégeant au niveau des quadrants internes et empiétant sur les quadrants externes, sans adénopathies axillaires le tout évoquant un abcès mammaire. L'exploration échographique (B) a trouvé une volumineuse masse solidokystique à composante liquidienne prédominante finement échogéne avec des cloisons épaisse et des bourgeons mesurant jusqu'à 46mm classée ACR4. Une ponction a ramené un liquide vert grisâtre envoyé pour étude bactériologique et une antibiothérapie à base de quinolones a été débutée. Le contrôle au bout de 3 jours a noté la diminution des signes inflammatoires, et l'étude bactériologique du liquide n'a trouvé aucun germe. A 15 jours d'antibiothérapie les signes inflammatoires ont disparu et le volume de la masse a discrètement diminué d'ou la décision de kystectomie. Laquelle a été réalisée avec énucléation facile (C), et à notre surprise l'étude histologique a conclu à un adénofibrome kystisé (D).

**Figure 1:**
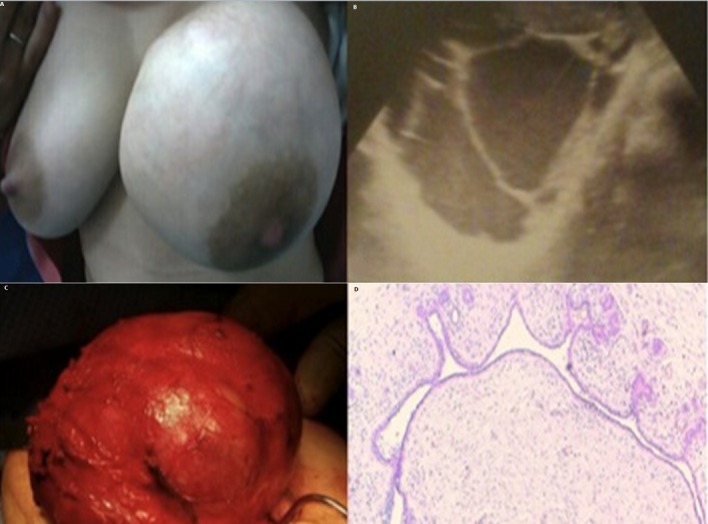
Un adénofibrome kystisé se révélant par un tableau de mastite infectieuse en post partum: A) sein gauche augmenté de volume avec des signes inflammatoires; B) aspect échographique d’une image solidokystique à composante liquidienne prédominante classée ACR 4; C) aspect per opératoire de la masse après son énucléation; D) aspect histologique d’un adénofibrome du sein

